# Xerostomia and Its Cellular Targets

**DOI:** 10.3390/ijms24065358

**Published:** 2023-03-10

**Authors:** Yoon-Jung Kim

**Affiliations:** Department of Physiology and Neuroscience, Dental Research Institute, Seoul National University School of Dentistry, Seoul 03080, Republic of Korea; agi@snu.ac.kr

**Keywords:** 1,4,5-trisphosphate receptor, aquaporin 5, G-protein-coupled receptors, intracellular calcium, parasympathetic nerves, store-operated Ca^2+^ entry, xerostomia

## Abstract

Xerostomia, the subjective feeling of a dry mouth associated with dysfunction of the salivary glands, is mainly caused by radiation and chemotherapy, various systemic and autoimmune diseases, and drugs. As saliva plays numerous essential roles in oral and systemic health, xerostomia significantly reduces quality of life, but its prevalence is increasing. Salivation mainly depends on parasympathetic and sympathetic nerves, and the salivary glands responsible for this secretion move fluid unidirectionally through structural features such as the polarity of acinar cells. Saliva secretion is initiated by the binding of released neurotransmitters from nerves to specific G-protein-coupled receptors (GPCRs) on acinar cells. This signal induces two intracellular calcium (Ca^2+^) pathways (Ca^2+^ release from the endoplasmic reticulum and Ca^2+^ influx across the plasma membrane), and this increased intracellular Ca^2+^ concentration ([Ca^2+^]_i_) causes the translocation of the water channel aquaporin 5 (AQP5) to the apical membrane. Consequently, the GPCR-mediated increased [Ca^2+^]_i_ in acinar cells promotes saliva secretion, and this saliva moves into the oral cavity through the ducts. In this review, we seek to elucidate the potential of GPCRs, the inositol 1,4,5-trisphosphate receptor (IP_3_R), store-operated Ca^2+^ entry (SOCE), and AQP5, which are essential for salivation, as cellular targets in the etiology of xerostomia.

## 1. Introduction

Saliva is essential for maintaining a healthy oral environment and overall health. The salivary glands regulate salivation according to the surrounding environment and circumstances, and various factors can affect the volume and composition of saliva. Xerostomia (dry mouth), defined as a subjective feeling of oral dryness [1], is a term derived from the Greek “xeros” (ξηρός), meaning “dry”, and “stoma” (στόμα), meaning “mouth”. Xerostomia results in decreased salivary flow and changes in the composition of saliva. This condition has various causes and is affected by the function of the salivary glands. However, a dry sensation in the mouth can also be observed in individuals with normal salivary gland function [2,3]. The main causes of xerostomia are aging, radiation to the head and neck, and Sjögren’s syndrome; however, the most common cause is drug-induced xerostomia, which is associated with more than 400 different drugs. Xerostomia decreases normal saliva function, which increases the occurrence of bad breath, dental caries, and dental erosion and can decrease quality of life due to issues such as food intake problems and depression. The estimated prevalence of persistent xerostomia varies between 10% and 50%, with a conservative estimate of 20% in the general population. It is also more commonly found in women (up to 30%) and older adults (up to 50%) [2,4,5,6], and medications and diseases, as well as aging itself, are generally considered to be important factors [7,8]. However, there is no permanent solution approved by the US Food and Drug Administration for salivary gland hypofunction and resultant xerostomia [9,10,11]. This is also why more research on the mechanism by which xerostomia occurs is needed. Herein, we summarize the mechanisms of salivation at the cellular level and targeted studies of xerostomia.

## 2. Unidirectional Movement of Fluid in the Salivary Glands

### 2.1. Structure of the Salivary Glands

Saliva performs a variety of functions essential for oral and systemic health. These include moisturizing and lubricating the mouth; enabling gustatory and olfactory sensation; protecting the teeth and oropharyngeal mucosa; facilitating speech articulation; allowing mastication, swallowing, and digestion; and maintaining a balanced microbiome [12,13,14,15,16,17,18,19]. The salivary glands, which are exocrine glands, secrete saliva, which is a mixture of proteins and fluids, into the mouth. A healthy adult produces between 0.5 and 1.5 L of saliva per day, 90% of which is produced by the three main salivary glands: parotid (PAR), submandibular (SM), and sublingual (SL) glands [20,21].

Most of the secretion from the PAR glands occurs in response to stimuli, while the SM and SL glands are responsible for the majority of unstimulated saliva production [22]. These glands differ in the types of secretion they produce: the PAR glands produce a serous, watery secretion; the SM glands produce a mixed serous and mucous secretion; and the SL glands secrete saliva that is predominantly mucous in character [22]. One striking example of a gland-specific expression is salivary amylase, which shows abundant expression at the protein level in the PAR and SM glandular tissue while being virtually absent in the SL glands [23]. This functional specialization of the adult salivary glands occurs during late-stage development [23]. In 2020, a new set of salivary glands, called the tubarial glands, was suggested as a fourth pair of salivary glands; these glands are situated posteriorly in the nasopharynx [24]. In addition, hundreds of minor salivary glands are distributed throughout the oral cavity, among which glands in the lower lip are easily biopsied and used clinically to diagnose Sjögren’s syndrome. Some studies have obtained RNA-seq data that suggest that cellular heterogeneity within gland types underlies gland-specific protein secretions [23].

The secretion of saliva from the three main salivary glands into the oral cavity occurs through their respective ducts. Stensen’s duct pierces the buccinator and connects the PAR gland to the buccal mucosa adjacent to the maxillary second molar [25,26]. Wharton’s duct is the main excretory duct of the SM glands and arises from the smaller, deep lobe inferior to the mucosa of the floor of the mouth and opens into the oral cavity under the tongue by the lingual frenulum at the SL caruncle [25,26]. The SL gland drains through a series of short ducts, all of which open into the floor of the mouth and are collectively termed the ducts of Rivinus [25,26].

### 2.2. Polarized Acinar Cells and Support Cells

To understand the mechanism of salivation, it is important to understand the structure of the cell level constituting the salivary gland. The salivary glands are composed of various epithelial cells, including acinar cells, which produce saliva; ductal cells, which transport saliva to the oral cavity; and myoepithelial cells, which facilitate the secretion of saliva [22].

Acini are formed by clusters of several pyramidal secretory cells, acinar cells, and are identified by the expression of markers, such as the water channel aquaporin 5 (AQP5) and the transcription factor muscle, intestine, and stomach expression 1 (Mist1) [27,28,29,30]. These cells can be serous, mucous, or seromucous, depending on the nature of their secretions and are present in relative proportions varying between glands and species [31,32,33]. Acini are linked to the lateral membrane through tight junctions formed with adhesion molecules, and this structure serves to prevent lateral movement of membrane proteins between the apical and basolateral membranes, contributing to cell polarity [34,35,36]. In the salivary glands, tight junctions permit unidirectional salivary secretion and maintain a cellular barrier between blood and tissue fluid [36].

The ductal system of the salivary glands serves as a conduit to modify the electrolyte content of saliva and to transport secretions to the oral cavity. In general, acinar cells secrete an isotonic plasma-like fluid, which is deposited in the lumen [37,38,39]. As it passes through the ductal system, saliva is progressively transformed into a hypotonic solution by the selective reabsorption of certain ions [40,41]. The intercalated ducts (IDs), which are the parts of an exocrine gland leading directly from the acinus to striated ducts (SDs), are formed from a single layer of cuboidal cells with a central nucleus and small secretory granules containing lysozyme and lactoferrin [22,25,33]. The SDs, which are lined by a long columnar epithelium with a central nucleus, participate in bidirectional transport and reabsorption of electrolytes and are characterized by numerous mitochondria forming cytoplasmic folds or striations in the basolateral membrane [25,33]. Along with the IDs, these function to modify salivary fluid by secreting bicarbonate (HCO_3_^−^) and potassium (K^+^) and reabsorbing sodium (Na^+^) and chloride (Cl^−^), making the saliva hypotonic. They compose most of the duct systems in the major salivary glands [33,42] and drain into interlobular ducts situated between the lobules of the gland.

Myoepithelial cells surround acinar cells and are sometimes found around the IDs [43]. These cells are smooth muscle epithelial cells characterized by the expression of contractile proteins [44]. Thus, these are essential for the contractile process around acinar cells to promote salivary excretion in response to nerve stimulation [45,46,47].

## 3. Salivation by G-Protein-Coupled Receptor (GPCR)-Mediated Intracellular Calcium (Ca^2+^) Signaling

### 3.1. Mode of Action of Salivation

**Figure 1:**
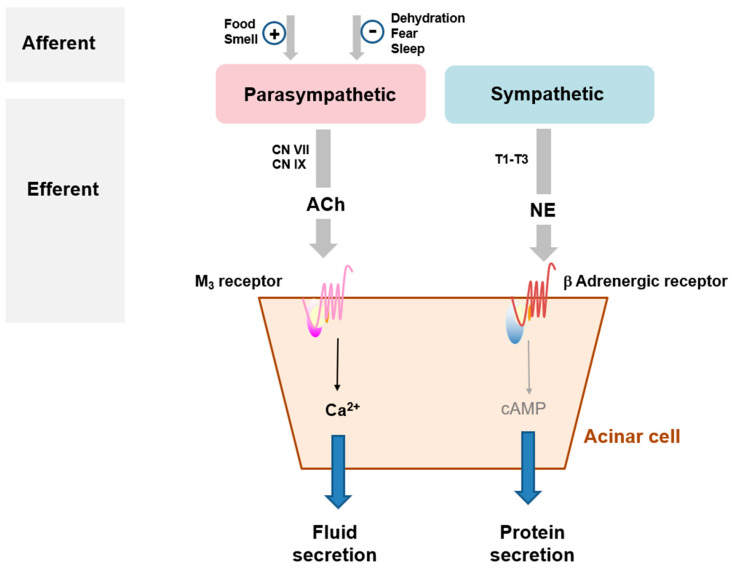
Neural control of salivation. Stimuli, such as food, smells, and fear, are integrated into the solitary nucleus in the medulla through the afferent pathway. Parasympathetic efferent pathways from the SL and SM glands originate from the facial nerve (VII), and the pathway to the PAR gland originates from the glossopharyngeal nerve (IX). Fluid and electrolyte secretion is activated by the binding of acetylcholine (ACh) to M_3_ subtype muscarinic ACh receptors (M_3_ mAChRs). Protein secretion is activated by the binding of norepinephrine (NE) to β adrenergic receptors. cAMP, cyclic adenosine monophosphate; CN, cranial nerve; T1–T3, thoracic segments.

The salivary response to seeing or even imagining sour food occurs because the salivary glands in the oral system receive strong neural input. Salivation is primarily under the control of the autonomic nervous system and is regulated by neurotransmitters and hormones [41,48,49]. Fluid secretion is initiated primarily by the binding of neurotransmitters released from parasympathetic nerves to a specific GPCR. Here, an increase in the intracellular Ca^2+^ concentration ([Ca^2+^]_i_) stimulated by neurotransmitter–GPCR binding in acinar cells is a major trigger for salivation [50,51]. Protein secretion from the salivary glands is regulated primarily by intracellular 3′,5′-cyclic adenosine monophosphate (cyclic AMP) via sympathetic nerves [50]. Cyclic AMP signaling contributes to digestion by lubricating food when chewing and swallowing, and it exerts important antiviral and antibacterial effects on oral tissues (Figure 1).

### 3.2. GPCRs as Keys for Cell-to-Cell Communication

Salivary gland cells are non-excitatory cells that lack voltage-sensitive channels and communicate with other cells, such as neurons, through GPCRs. Thus, saliva secretion begins with the activation of specific GPCRs on released neurotransmitters [38,52,53], and GPCR-mediated signaling and salivary gland dysfunction are closely related [51,52,53,54]. mAChRs, specifically M_3_ subtype mAChRs (M_3_ mAChRs), are essential for the parasympathetic control of salivation in mice [53,55]. In the case of M_1_ or M_3_ single-knockout (KO) mice, the amount of pilocarpine-induced salivation was greatly reduced at a low concentration (1 mg/kg), but there was no significant difference compared with wild-type mice at a high concentration (15 mg/kg). In the case of double-KO mice, salivation was completely lost regardless of the pilocarpine concentration [55]. Carbachol (CCh)-induced increased [Ca^2+^]_i_ in SM gland cells showed little difference in M_1_ KO mice compared with control mice but was greatly reduced in M_3_ KO mice and completely lost in double-KO mice [53]. Two-dimensional Ca^2+^ imaging analysis in response to CCh in individual acinar cell clusters suggested that the distribution of M_1_ in SM gland acini is not ubiquitous and that some acinar cells express M_1_ at a high level [53]. mAChRs are expressed at different levels of abundant subtypes by a gland or species type, and human labial glands express M_1_, M_3_, and M_5_ mAChRs [56]. Interestingly, upregulation of M_3_, M_4_, and M_5_ expression was observed in samples from patients with Sjögren’s syndrome [56,57].

In addition to mAChRs, a series of GPCRs, including GPR39, histamine H_1_ receptor, sphingosine–1–phosphate (S1P) receptor, bradykinin receptor, and P2Y_2_ receptor (P2Y_2_R), has been investigated to identify salivary gland-related functions [52,58,59,60,61]. GPR39 is a type of GPCR with zinc as a ligand and is expressed in human SM gland tissues [52]. Interestingly, this study showed that salivary secretion significantly increased when human subjects gargled with a zinc-containing solution. These effects were observed both in a normal group and various hyposalivation groups, including a group of patients with Sjögren’s syndrome [52]. In primary cultured cells of the human SM gland and human SM gland (HSG) cell lines, histamine increased the [Ca^2+^]_i_, and the histamine H_1_ receptor was expressed [59]. Other types of receptors, including S1P 1, 2, 3, and 4 receptors, are expressed in human SM gland cells [60]. S1P triggers Ca^2+^ signaling and induces the expression of interleukin 6 (IL-6) and Fas, which are known to be involved in a Sjögren’s syndrome-related apoptotic pathway [60]. Bradykinin B2 receptors are expressed in human SM gland tissue, and treatment with bradykinin induces intracellular Ca^2+^ signaling [61]. P2 purinergic receptors for extracellular nucleotides, including P2Y_1_ and P2Y_2_, are expressed in rat SM acinar and ductal cells and are involved in intracellular Ca^2+^ signaling [62,63,64,65]. In particular, due to its ability to stimulate water transport across epithelial cell membranes, the P2Y_2_R agonist diquafosol has undergone human clinical trials for the treatment of dry eye disease and is currently approved for human use in South Korea and Japan under the trade name Diquas [66,67,68].

### 3.3. Stimulation of Fluid Secretion by GPCR-Mediated Increases in [Ca^2+^]_i_ in Acinar Cells

The salivary gland cells regulate their secretions through neurotransmitter-generated Ca^2+^ signaling. This is regulated by autonomic sympathetic and parasympathetic stimuli. In particular, ACh secreted from parasympathetic nerves is known to be the most important salivary secretory factor in the salivary glands. Enhancing fluid secretion in the salivary glands requires a series of processes, including activation of membrane receptors, including mAChRs, through binding between neurotransmitters and specific GPCRs, increases in cytoplasmic [Ca^2+^]_i_, and stimulation of ion transport pathways. When there is an increase in [Ca^2+^]_i_ in the acinar cells, ion channel activity is regulated in various domains of the cells, the AQP5 channels are translocated to the apical membrane, and water secretion occurs.

**Figure 2:**
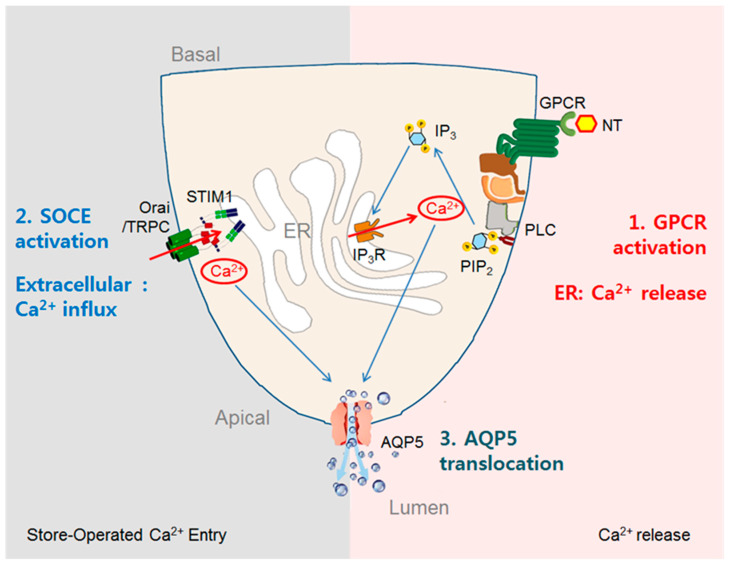
Ca^2+^ signal transduction and regulation of fluid secretion in salivary gland acinar cells. This figure shows the key signaling events and components involved in the regulation of fluid secretion in salivary gland cells: Fluid is activated by the binding of ACh to subtype M_3_ mAChRs. Binding activates a GPCR, and the target enzyme is phospholipase C (PLC), which splits phosphatidylinositol 4,5-bisphosphate (PIP_2_) into diacylglycerol (DAG) and inositol 1,4,5-trisphosphate (IP_3_). IP_3_ acts by binding to the IP_3_R on the endoplasmic reticulum (ER) and releasing the Ca^2+^ stored there. (First step; Ca^2+^ release from the ER). Stromal interaction molecule–1 (STIM1) in the ER membrane acts as a Ca^2+^ sensor, causing structural changes when the ER is depleted, and forms store-operated Ca^2+^ entry (SOCE) with Orai channels or transient receptor canonical (TRPC) channels expressed in the plasma membrane of acinar cells. This leads to an influx of extracellular Ca^2+^ (second step; Ca^2+^ influx via the plasma membrane), followed by the translocation of AQP5 at the apical membrane (third step). These increases in [Ca^2+^]_i_ as a result of neurotransmitter–GPCR binding induce the regulation of ion transport, the production of an osmotic gradient, and the flow of water.

Two steps (Ca^2+^ release from the ER and Ca^2+^ influx via the plasma membrane) are required to increase [Ca^2+^]_i_ in the salivary glands and maintain the saliva secretion state (Figure 2). Stimulation of GPCRs, such as mAChRs and α1-adrenergic receptors, results in phospholipase C (PLC) activation. Sequentially, PLC hydrolyzes phosphatidylinositol 4,5-bisphosphate (PIP_2_) into inositol 1,4,5-trisphosphate (IP_3_) and diacylglycerol (DAG). The initial increase in [Ca^2+^]_i_ after external stimulation of acinar cells is triggered by the release of Ca^2+^ from the ER via the binding of cytosolic IP_3_ and IP_3_ receptors (IP_3_Rs) at the ER membrane. In exocrine gland cells, IP_3_R2 and IP_3_R3 are concentrated in the apical pole of the cell [69,70], and in response to external stimuli, [Ca^2+^]_i_ increases in the apical region, spreads to the basal pole, and activates various ion channels and transporters to coordinate fluid secretion [41,71,72,73]. Pilocarpine-induced salivation is seriously impaired in IP_3_R2 and IP_3_R3 double-KO mice, which lose weight and die within four weeks without wet mash food [74].

The IP_3_-induced increase in [Ca^2+^]_i_ from the ER is essentially transient in the absence of extracellular Ca^2+^ [49]. Subsequent activation of store-operated Ca^2+^ entry (SOCE) converts the transient increase in [Ca^2+^]_i_ into a sustained increase essential for long-term salivation [49] (Figure 2). Ca^2+^ depletion of the ER initiates the activation of SOCE [49,72]. Stromal interaction molecule–1 (STIM1) at the ER membrane acts as a Ca^2+^ sensor, which causes a conformational change when the concentration of Ca^2+^ in the ER is lowered and forms SOCE with Orai channels or transient receptor potential canonical (TRPC) channels expressed in the plasma membrane of acinar cells [75,76,77,78,79]. This process allows extracellular Ca^2+^ to enter the cell. Orai1 is the best characterized among the members of the Orai channel family, and it generates a highly Ca^2+^-sensitive, inwardly rectifying Ca^2+^ current when activated by STIM1 [77,79,80]. TRPC channels function as Ca^2+^-permeable nonselective cation channels, and all members are activated in response to PIP_2_ hydrolysis stimulated by neurotransmitters [81,82]. TRPC1 is an essential channel for salivary gland function, and lack of this channel results in an attenuation of store-operated Ca^2+^ current and a significant loss of fluid secretion [75,76]. Perturbation of SOCE activity is thought to be an important toxic mechanism because SOCE is required for the maintenance of a constant intracellular Ca^2+^ pool and GPCR signaling [83].

[Ca^2+^]_i_ plays a particularly important role in regulating K^+^, Na^+^, and Cl^−^ fluxes and salivary secretion in acinar cells. Salivation is initiated when an increased [Ca^2+^]_i_ activates K^+^ and Cl^−^ channels and is maintained as long as this is sustained [41,49]. For fluid secretion, transepithelial transport of Cl^−^ from the basolateral to the apical side of the cell is required, and Na^+^ flux through the tight junction leads to the accumulation of NaCl in the lumen, resulting in water secretion through the AQP5 channel expressed in the apical membrane with the generated osmotic gradient [41,49]. In addition, the cell enters a hyperpolarized state through K^+^ efflux via the apical and basolateral membranes to support fluid secretion. [Ca^2+^]_i_ is maintained at approximately 50–100 nM in resting cells, which is less than the threshold required to activate the K^+^ and Cl^−^ channels [49].

Aquaporins (AQPs), a family of transmembrane channel proteins, are responsible for water transcellular permeability in most living organisms [84]. In mammals, AQPs are largely divided into classical AQPs permeable only to water (AQP1, AQP2, AQP4, AQP5, AQP6, and AQP8) and aquaglyceroporins permeable to small solutes such as glycerol and urea in addition to water (AQP3, AQP7, AQP9, and AQP10) [85,86,87]. In human salivary glands, AQP1 expression is restricted to the vascular endothelium and myoepithelial cells surrounding acini [29,88,89]. In acinar cells, AQP3, AQP4, and AQP5 are expressed, and the subcellular localization enriched in each is different [29,88,89,90,91]. Expression of AQP3, an aquaglyceroporin, has been detected at the apicolateral membrane of serous cells and at the apical pole of mucous acinar cells [29], while AQP4 expression has been localized to the basal membrane of acinar cells [90]. AQP5 is predominantly expressed at the apical membrane in acinar cells and not expressed in mature ducts [29,88,89,91]. AQP5 plays a key role in the secretion of saliva, and briefly, activation of subtype M_1_ and M_3_ mAChRs leads to an increased [Ca^2+^]_i_ that induces AQP5 trafficking to the acinar apical membrane [92,93,94,95]. Parasympathectomy has been found to significantly decrease salivary AQP5 protein levels without affecting mRNA levels [96], through a post-transcriptional mechanism involving protein degradation [91,97]. The neural signal via the parasympathetic nerve innervating the SM glands, i.e., the chorda tympani nerve, has been suggested to be responsible for maintaining a certain degree of AQP5 expression [96]. Sympathetic activation leads to increased cAMP and a subsequent increase in RNA levels and the translocation of AQP5 to the cell apical membrane [91,98,99]. AQP5 KO mice presented an approximately 60% reduction in pilocarpine-induced saliva secretion, indicating that AQP5 plays a major role in water permeability in acinar cells and saliva secretion [94,95].

## 4. Increasing Prevalence of Xerostomia

The majority of the 550,000 patients who undergo radiation treatment for head and neck cancer annually and more than 4 million patients with Sjögren’s syndrome worldwide suffer from salivary gland dysfunction [100]. More than 400 different drugs, including antidepressants, antipsychotics, antihistamines, antihypertensives, and others, are also major causes [8,101,102,103,104,105]. Aging itself is also a cause of xerostomia, but its incidence continues to increase as polypharmacy among the elderly increases [106,107]. The combined number of multiple drugs greatly increases xerostomia, and among them, long-term use of drugs, including psychotropic drugs, causes chronic xerostomia and significantly reduces the quality of life. However, there is currently no permanent curative therapy, and the general management approach is directed at palliative treatment for the relief of symptoms and prevention of oral complications [104,108]. Systemic sialagogues approved by the Food and Drug Administration (FDA) for salivary gland dysfunction include muscarinic agonists, such as pilocarpine and cevimeline, as mimic form parasympathetic nerve action [109,110]. However, since mAChRs are widely expressed in various organs and tissues of the body, their use has various side effects [9,10,11].

### 4.1. Xerogenic Drugs as the Most Common Cause of Xerostomia

Drugs are the most frequent cause of dry mouth [111] and are called xerogenic drugs [112] (Table 1). These include antidepressants, antiemetics, antihistamines, antihypertensives, antipsychotics, appetite suppressants, anxiolytics, bronchodilators, cardiovascular agents, and muscle relaxants [8,101,102,103,104,105]. Some cause subjective dry mouth symptoms, and many can cause decreased salivation. Although there appear to be several mechanisms by which drugs can cause dry mouth, few have been subjected to in-depth scientific investigation [111]. Early antidepressants, including tricyclic antidepressants (TCAs), unfortunately block histaminergic, cholinergic, and α1-adrenergic receptor sites, resulting in a variety of adverse drug reactions (ADRs), including dry mouth as well as weight gain, constipation, drowsiness, and dizziness [111]. Muscarinic receptor antagonists, which are recommended as first-line therapy for an overactive bladder, can also cause dry mouth [111].

The likelihood of xerostomia increases with the total number of medications taken, regardless of whether the individual medications cause dry mouth. There are many other types of receptors for endogenous substances in the salivary glands that can be causes of drug-induced dry mouth, but anticholinergic actions contribute significantly. Synergistic effects of drug combinations contribute to xerostomia; in addition, although saliva flow does not necessarily decrease with age, older people are more likely than younger people to develop xerostomia due to the increased prevalence of chronic conditions requiring pharmacological treatment [106,113]. Drug-induced xerostomia is usually reversible, but the conditions for which these drugs are prescribed are often chronic [114]. Long-term treatment for schizophrenia with conventional phenothiazine antipsychotics is commonly associated with ADRs, including dry mouth [111]. Cardiovascular medications in hospitalized elderly patients and respiratory diseases in the elderly outpatients are the main factors for xerostomia, but the use of psychiatric drugs is also the strongest explanatory factor for all patients [7].

### 4.2. Systemic Diseases and Salivary Gland Disorders That Compromise Glandular Tissue Integrity

Systemic diseases that affect the salivary glands can cause salivary dysfunction, resulting in xerostomia [115] (Table 2). Among these, severe hyposalivation is frequently caused by Sjögren’s syndrome, a chronic autoimmune disease [116]. Excessive infiltration of inflammatory cells, resulting in increased production of cytokines and degradation of tissue proteins, destroys the acinar cells and interferes with salivary synthesis, resulting in dysfunction of the salivary glands [117]. Multiple innate immune pathways, including the nuclear factor-κB pathway, are likely dysregulated in the salivary gland epithelium in Sjögren’s syndrome [118]. Thus, both generic and oral health-related quality of life are poor in these patients [116]. Intraoral imaging using ultra-high frequency ultrasonography, a recently introduced diagnostic technique, plays an increasingly important role in small salivary gland biopsy and subsequent focal scoring, which are critical in the diagnostic workup of this disease [119,120].

Radiation therapy is an important treatment for patients with head and neck cancer. However, the salivary glands are often inadvertently irradiated and damaged because they are within the irradiated area [121]. Cumulative exposure to radiation causes excessive destruction of saliva-producing acini and reduced salivary flux [122,123]. Many advances in the management of radiation-induced salivary gland hypofunction still only offer partial protection [123]. Decreased salivation in these patients has serious consequences for oral somatosensory alterations that can lead to malnutrition [124].

Salivary gland tumors are uncommon, representing less than approximately 5% of all cancers of the head and neck [125]. Surgery is usually performed to remove the tumor and surrounding tissue, but this can also lead to reduced salivary gland function and xerostomia [126].

Both unstimulated and stimulated salivation decrease with age in humans [127]. Histological studies have demonstrated that with age, the mean volume of acini decreases by approximately 30% in the SM glands, nearly 25% in the SL glands, and approximately 12% in the PAR glands [107]. In addition, the number of terminal deoxynucleotidyl transferase dUTP nick end labeling (TUNEL)-positive apoptotic cells in the SM glands has been found to increase with age, suggesting that cell turnover and cellular changes contribute to age-dependent salivary gland dysfunction [128].

### 4.3. Current Palliative Care and Pharmacological Therapies

Systemic sialagogues mimic the neural signals that stimulate saliva production in the epithelium. Anterior sialagogues mentioned by the FDA and the National Institute for Health and Care Excellence include pilocarpine and cevimeline [109,110], which stimulate salivary tissue. Pilocarpine is a nonselective muscarinic agonist with a relatively high affinity for CNS muscarinic receptors and cevimeline has a higher affinity for M_1_ and M_3_ mAChRs. Head and neck radiation-treated patients with established hyposalivation respond minimally to systemic sialagogues [129]. In addition, since mAChRs are widely expressed in the body, the use of these drugs is associated with various side effects such as nausea, diarrhea, increased urinary frequency, excessive sweating, cutaneous vasodilation, bronchoconstriction, hypotension, and bradycardia [9,10,11].

Saliva substitutes or artificial saliva are often prescribed to temporarily relieve xerostomia. They generally contain a thickening agent and have protective properties but poor antibacterial and antifungal properties [130]. Most saliva substitutes aim to mimic the rheological properties of saliva and consist of rheological modifiers such as xanthan and guar gums, as well as carboxymethyl cellulose or hydroxyethyl cellulose, glycerol, mucins, electrolytes, preservatives, and sweeteners. Saliva substitutes have not yet been able to mimic the antibacterial properties of saliva [130].

Mouth rinses, mouthwashes, and toothpaste can provide short-term relief from dry mouth and keep the patient’s mouth, teeth, and gums healthy. Such mouth rinses can greatly increase saliva volume and improve pH buffering [131]. However, similar to saliva substitutes, these treatments only treat symptoms for a short period of time (up to 4 h) and do not solve the underlying clinical problem.

## 5. Conclusions

Normal salivation is important for oral and overall health and wellbeing. Xerostomia is clearly a problem faced by an increasing proportion of the population. Accordingly, elucidating the mechanisms leading to the loss of salivary secretion and those involved in functional rescue should be a major focus of salivary research in the future. Acinar cells are polarized and are responsible for the unidirectional movement of fluid. Cellular heterogeneity among gland types derived from RNA-seq data suggests that various cells are coordinated within the salivary gland. Further elucidating this diversity will require a detailed study of the components of each cell unit. Importantly, saliva secretion is precisely regulated by GPCR-mediated intracellular Ca^2+^ signaling. Therefore, it is expected that regulators controlling the exocrine intracellular Ca^2+^ mechanism contribute directly to salivation. Key components involved in intracellular Ca^2+^ signaling include GPCR, IP_3_R, SOCE, and AQP5, all of which can be targets of medication or diseases. Conversely, acinar cell-specific GPCR agonists, such as mAChR and P_2_Y2R agonists, are potential therapeutic candidates that can regulate intracellular Ca^2+^ signaling at specific cellular units.

## Figures and Tables

**Table 1:** Xerogenic drugs.

Classification	Drugs
Analgesics	Opioids, pregabalin, tramadol.
Anticonvulsants	Carbamazepine, gabapentin, lamotrigine.
Antidepressants	Tricyclics (e.g., amitriptyline, clomipramine, desipramine, doxepin, imipramine, nortriptyline, protriptyline, trimipramine), selective serotonin reuptake inhibitors (e.g., citalopram, escitalopram, fluoxetine, fluvoxamine, paroxetine, sertraline), serotonin and noradrenaline reuptake inhibitors (e.g., venlafaxine), and atypical antidepressants (e.g., bupropion, duloxetine, mirtazapine, trazodone).
Antiemetics	Buclizine, cyclizine, dimenhydrinate, meclizine, metocloopramide, prochloperazine, scopolamine, thiethylperazine, trimethobenzamide.
Antihistamines	First-generation antihistamines (carbinoxamine, clemastine dexchlorpheniramine, dimenhydranate, diphenhydramine, hydroxyzine, meclizine, promethazine), and second-generation antihistamines (cetirizine, desloratadine, fexofenadine, levocetirizine loratadine).
Antihypertensives	α-agonists (clonidine, guanabenz, guanfacine, methldopa), β-blockers (acebutolol, atenolol, bebivolol, betaxolol, bisoprolol, carvedilol, esmolol, labetalol, metoprolol, nadolol, penbutolol, pindolol, propranolol, stalol, timolol), diuretics (bumetanide, furosemide, torsemide), Ca^2+^ channel blockers (amlodipine, diltiazem, felodipine, isradipine, nifedipine, nimodipine, verapamil), and angiotensin-converting enzyme inhibitors (benazepril, captopril, enalapril, fosinopril, lisinopril, moexipril, perindopril, quinapril, ramipril, trandolapril).
Antiparkinsonian	amantadine, benztropine, bromocriptine, carbidopa, entcapone, levodopa, pramipexole, rasagiline, ropinirole, selegiline, trihexyphenidyl.
Antipsychotics	Typical antipsychotics (e.g., chlorpromazine, fluphenazine, haloperidol, loxapine, perphenazine, pimozide, trifluoperazine) and atypical antipsychotics (e.g., aripiprazole, amisulpiride, clozapine, olanzapine).
Appetite suppressants/stimulants	Benzphetamine, diethylpropion, phentermine, phendimetrazine, sibutramine.
Anxiolytics	Alprazolam, chlordiazepoxide, clorazepate, diazepam, doxepin, hydroxyzine, lorazepam, meprobamate, oxazepam, prazepam.
Bronchodilators	Albuterol, eformoterol, ipratropium, metaproterenol, pirbuterol, salbutamol, salmeterol, tiotropium, umeclidinium.
Cardiovascular agents	Atenolol, clonidine, metoprolol, prazosin.
Muscle relaxants	Baclofen, cyclobenzaprine, orphenadrine.

**Table 2:** Systemic diseases associated with xerostomia.

Systemic Diseases
Sjögren’s syndrome
Systemic lupus erythematosus
Diabetes (type 1 and type 2)
Viral infection (e.g., human immunodeficiency virus, hepatitis C virus, and human T-lymphotropic virus type 1)
End-stage renal disease
Primary biliary cirrhosis
Ectodermal dysplasia
Graft-versus-host disease
Sarcoidosis

## Data Availability

Not applicable.
